# Teicoplanin-Induced Anaphylactic Reaction in Children: A Case Report

**DOI:** 10.3390/pharmacy6040110

**Published:** 2018-10-09

**Authors:** Godwin Oligbu, Leila Ahmed

**Affiliations:** 1 Paediatric Department, Northwick Park Hospital, London HA1 3UJ, UK; leila.ahmed1@nhs.net; 2 Paediatric Infectious Diseases Research Group, Institute for Infection and Immunity, St. George’s, University of London, London SW17 0RE, UK

**Keywords:** teicoplanin, anaphylaxis, children

## Abstract

Teicoplanin is now increasingly used as a first-line prophylactic therapy for major surgical procedures, treatment of methicillin-resistant Staphylococcus aureus infections and for those with reported penicillin allergy. Teicoplanin is rarely associated with anaphylaxis and there is limited information on the prevalence of teicoplanin-induced perioperative anaphylaxis. Here, we describe a case of a 12-year-old child with teicoplanin-induced anaphylaxis peri-operatively.

## 1. Introduction

Teicoplanin is a semisynthetic glycopeptide antibiotic with a spectrum of activity similar to vancomycin in inhibiting bacterial cell wall synthesis. It is an effective agent against gram-positive microorganisms. With increasing evidence that antibiotic prophylaxis during surgical procedure prevent post-operative infection, Teicoplanin is now used both as first-line prophylactic therapy for some major surgeries, particularly orthopaedic procedures, and is often the chosen therapy for those reporting penicillin allergy. It is also increasingly used in the treatment of methicillin-resistant Staphylococcus aureus infections [1,2,3,4,5]. Anaphylaxis to Teicoplanin was previously felt to be very rare. However, recently, there is increasing recognition of allergies to this agent. In a recent UK wide survey of anaesthetists’ perspectives and experiences of severe perioperative anaphylaxis, of 11,104 anaesthetists, only about 5% of the respondents perceived teicoplanin as causing perioperative anaphylaxis [6]. The prevalence of teicoplanin-induced perioperative anaphylaxis is therefore of clinical consequence and it is important that clinicians and particularly anaesthetists are aware of the possible anaphylactic reaction associated with its use [7].

Here, we describe a patient with bone deformity who developed anaphylactic reaction to teicoplanin peri-operatively. 

## 2. Case Summary

A 12-year-old previously healthy, with no known or significant family history of allergy, was routinely admitted for the corrective bone surgery. She received 2% Propofol, Remifentanil and 30 mg of intravenous (IV) Ketamine bolus along with prophylactic 600 mg IV Teicoplanin as a 20 min infusion within a short period of time for induction of her anaesthesia. Her surgery was abandoned due to anaphylactic shock within minutes of induction. She became profoundly hypotensive (BP 44/25 mmHg) with a weak and thready pulse. She also developed facial angioedema. She was treated with fluid boluses, four doses of 25 mcg of adrenaline, 20 mg of IV chlorphenamine and 200 mg hydrocortisone.

Following discharge, she reported initial forgetfulness for three days but this quickly resolved and she was able to return to school and activities as normal. However, she continued to have facial eczema and localised skin reaction around her cannula site for up to 6 weeks.

She was investigated with repeat intradermal testing (IDT) for the agents used preoperatively. IDT for Teicoplanin was found to be positive (1:10 dilution, repeated twice) and delayed positive on a third occasion where she developed persistent swelling of her arm for days following the test. IDT for fentanyl was negative on (1:10, 1:100). In addition, she had intranasal fentanyl challenge as well as a graded IV fentanyl challenge to ensure that she could have opiates intra-operatively. She was also noted to be equivocal to vancomycin (1:10,000) on two occasions. Vancomycin was therefore avoided in her management. Assessment for possible latex sensitization was also negative.

Having successfully passed a graded fentanyl challenge, she proceeded with her corrective bone surgery 6 months later for which she had Fentanyl and Rocuronium along with inhalational induction agents.

## 3. Discussion

IgE-mediated anaphylaxis due to glycopeptide antibiotics, such as vancomycin or teicoplanin, have been considered until recently, a rare phenomenon. Although reactions due to other glycopeptides such as vancomycin have been well described in the literature, our case appears to be the result of an IgE mediated anaphylaxis [8,9]. There was a good clinical history of anaphylaxis as described by Savic and colleagues [10]. This case in addition to others previously reported would suggest that teicoplanin allergy is more common than previously reported in the literature.

A recent case series of reactions to teicoplanin highlighted teicoplanin anaphylaxis as an emerging problem in anaesthetic allergy clinics, reporting seven definite cases from two UK centres and another from a tertiary orthopaedic hospital [10]. These studies estimate the rate of IgE-mediated anaphylaxis to be between 0.046% and 0.059% (equating to between 1:2088 and 1:1655) in 18,800–19,600 patients who received teicoplanin peri-operatively during the study period. In the study, there were 14 cases of suspected anaphylaxis attributed to the administration of teicoplanin and no fatality was reported [11]. In another recent UK wide 6th national audit on perioperative anaphylaxis collected in all NHS hospitals over a one year period, of the 36 patients who had anaphylaxis following teicoplanin, 2 died. Both were adults and with other underlying medical condition. Although, anaphylaxis from teicoplanin is life threatening, fatality in children is rare [12]. In addition, we observed an unequivocal finding of vancomycin (1:10,000) on 2 occasions suggesting the possibility of a cross-reaction to Teicoplanin. Hypersensitivity syndrome to both vancomycin and teicoplanin is rare but well documented [13]. 

Our case was confirmed by IDT. Although IDT for this agent has improved significantly in the last few years, there is nevertheless a small possibility of a false-positive as a result as the use of increasing concentrations of Teicoplanin, particularly for IDT, as it is known to cause non-specific skin reactions [7].

IgE mediated anaphylaxis to Teicoplanin is encountered in the perioperative setting more frequently than previously thought and as the clinical consequences could be devastating, there is a need for increased awareness by clinicians.
